# Fused Deposition Modeling and Characterization of Heat Shape Memory Poly(lactic) Acid-Based Porous Vascular Scaffold

**DOI:** 10.3390/polym15020390

**Published:** 2023-01-11

**Authors:** Li Zhang, Muhammad Hanif, Jiacheng Li, Abdul Hakim Shah, Wajid Hussain, Guotao Zhang

**Affiliations:** 1 Faculty of Mechanical Design and Vehicle Engineering, School of Mechanical Science and Engineering, Huazhong University of Science and Technology, Wuhan 430074, China; 2 Department of Physics, Khushal Khan Khattak University, Karak 27200, Pakistan; 3 Advanced Biomaterials and Tissue Engineering Centre, School of Biomedical Engineering, College of Life Sciences and Technology, Huazhong University of Science and Technology, Wuhan 430074, China

**Keywords:** shape memory polymers, solid isotropic material penalization method (SIMP), vascular scaffolds, thermodynamic properties, mechanical properties

## Abstract

Shape memory polymers have received widespread attention from researchers because of their low density, shape variety, responsiveness to the environment, and transparency. This study deals with heat-shape memory polymers (SMPs) based on polylactic acid (PLA) for designing and fabricating a novel porous vascular scaffold to treat vascular restenosis. The solid isotropic material penalization method (SIMP) was applied to optimize the vascular scaffolds. Based on the torsional torque loading of Hyperworks Optistruct and the boundary conditions, the topological optimization model of a vascular scaffold unit was established. Forward and reverse hybrid modeling technology was applied to complete the final stent structure’s assembly. The glass transition temperature for the present SMPs is 42.15 °C. With the increase in temperature, the ultimate tensile strength of the SMPs is reduced from 29.5 MPa to 11.6 MPa. The maximum modulus at room temperature was around 34 MPa. Stress relaxation curves show that the material classification is a “thermoset” polymer. The superb mechanical properties, the transition temperature of the SMPs, and the recovery ratio made it a feasible candidate for a vascular scaffold. A circular tube based on the shape memory polymers was presented as an example for analyzing the recovery ratio in an unfolding state. A higher recovery ratio was obtained at a temperature of 65 °C with a tube thickness of 2 mm. Finally, the proposed porous vascular scaffold was successfully fabricated, assessed, and compared with the original and previously developed vascular scaffolds. The proposed scaffold structure regains its initial shape with a recovery ratio of 98% (recovery temperature of 47 °C) in 16 s. The tensile strength, Young’s modulus, and bending strength of the proposed scaffold were 29.5 MPa, 695.4 MPa, and 6.02 MPa, respectively. The results showed that the proposed scaffold could be regarded as a potential candidate for a vascular implantation.

## 1. Introduction

**Figure 1:**
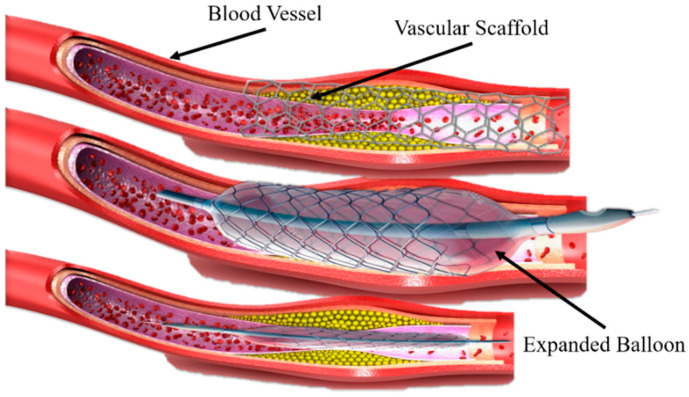
A vascular stent made of Ni-Ti shape memory alloy and balloon-assisted vascular implantation.

A new approach for printing innovative shape memory materials has emerged, which is the combination of three-dimensional (3D) printing and shape-changing materials (with time dimension), known as 4D printing [1]. Shape memory polymers (SMPs) have unique features of shape-changing behavior and functionality in response to the external environment [2,3,4]. Recently, SMPs have been widely used in many fields, such as biomedical devices [5], stents repairments [6], sensors and robotics [7], and so on. SMPs can be deformed to a temporary shape and regain their initial shape under the influence of various external environments or stimuli (light, heat, temperature, electric and magnetic field, etc.) by a phenomenon termed the shape memory effect (SME) [8,9]. Previously, Ni-Ti shape memory alloys (SMAs) were mainly used that can achieve a high-precision implantation and support vascular restenosis during a clinical operation under adjustable shape memory effect characteristics, diversified preparation methods [10,11] minor rebound stress, and slight surgery damage [12], as shown in Figure 1. However, the shortcomings of Ni-Ti SMAs are: (1) the auxiliary implantation of the balloon structure changes the vessel of the human body, and the difference between the scaffold structure and the cell micro-environment in the human body is further increased or decreased; (2) the long-term use of drugs can reduce the rejection of the scaffold by the human body, but the side effects will also hinder cell proliferation; and (3) the material is mainly based on metals, the biological tolerance is insufficient, and the later stage of rehabilitation requires a secondary surgery to remove the scaffold body and balloon structure, bringing a new pain and burden to patients. Therefore, the concept of biodegradable stents was proposed to overcome the above problems, mainly including PLA and PCL. These polymers can degrade after an implantation in the body without any operation and reduce body pain. Polymers have received widespread attention from researchers and worked continuously to improve materials. Compared with the SMAs, SMPs have a higher deformation strain, lower density and manufacturing cost, potential biodegradability, and processability [10].

Therefore, SMPs have potential applications in biomedical equipment, aerospace, textile, actuators, flexible electronic equipment, and other fields [13,14,15]. Polylactic acid (PLA) has a higher glass transition temperature, making it hard to adapt in the human body to realize the thermal-derived shape memory effect. Therefore, Zarek et al. (2016) have synthesized shape memory polymers based on polycaprolactone (PCL) for flexible electronic devices [16]. Considering the thermo-induced SMPs, a shape memory cycle typically includes three steps: (1) the sample is deformed at a specific deformed temperature (T_d_) higher than the glass transition temperature (T_g_) of the SMPs, resulting in a decrease in the conformational entropy of the polymer chain; (2) apply a load to deform the sample to a temporary shape and cool it to room temperature (lower than T_g_); and (3) again, reheat the SMPs sample above the transition temperature (recovery temperature) so that the polymer chain regain to their initial conformational state of high entropy, thereby returning to a permanent shape [2,3]. Various researchers have developed numerous SMPs to control the deformation, shape, and recovery behavior through a more convenient external stimuli, such as currents, magnetic fields, water, heat, etc. For instance, Rahmatabadi et al. (2022) have blended two different polymers and have synthesized a high-performance PLA-TPU biopolymer for medical purposes. Their mechanical properties, fracture toughness, and microstructures were evaluated [17].

Similar research was carried out for the tensile shape memory properties of Poly-Ethylene Terephthalate Glycol (PETG), which provides strong evidence at different parameters [18]. Weijian et al. (2022) have reviewed the scaffolds based on SMPs and compared their performance under a thermal environment [19]. The other stimuli, such as solvents [20], pH [21,22], and temperature [23], can further broaden the application of shape memory polymers in different specific areas. The water environment is pleasant, inexpensive, and environmentally friendly compared to other external stimuli, making water or humidity-induced SMPs ideal for use as intelligent biomedical devices [24]. Stents based on poly(l-lactide) (PLLA) and polycaprolactone are continuously growing in the industry. However, they have a weak mechanical performance (PCL) for biomedical products [25]. Investigating new SMPs with a thermal trigger operation and the high service life is one of the leading research directions in this field. SMPs are inevitably subjected to mechanical damage, such as accidental scratches or impacts. However, it has healing properties [26]. In addition, the shape recovery rate of thermoplastic SMPs generally decreases as the shape memory cycle increases, resulting in the degradation of the shape memory function [27]. These factors limit the reuse of thermoplastic SMPs. Therefore, there is a need to investigate new heat SMPs, which may be compatible with the soft tissues and provide superb mechanical and higher recovery ratios. Zhou et al. (2021) have synthesized a new star-polymer (s-PCL) modified by the acrylate end-group using a ring-opening polymerization technique. A novel framework was proposed to develop a 4D-printed scaffold for treating critical limb ischemia and the drug release [28]. A vascular scaffold is a biomedical device used to repair a blood vessel or open arteries or blockages [29,30]. In addition, the cell survival rate of implanted stent structures is determined by material selection, and the structural design pore distribution will affect the overall performance [31]. A novel study was conducted on PETG to check their self-morphing and memory behavior by considering the print parameters and physical aging as new post-treatment processes [32]. However, the current structural design of vascular stents is an array of simple configurations (rectangular, triangular, etc.).

This research investigated the PLA-based SMPs material, and their properties were assessed through several characterizations. The unique features of SMPs, through a thermal shape memory, which effect and shape the recovery ratio, were also characterized and verified. The influence of the recovery temperature and thickness on the recovery rate was studied. Finally, the application of SMPs in a vascular scaffold was presented. The proposed porous vascular scaffold was optimized based on solid isotropic material with a penalization (SIMP) technique. The stability, flexibility, and mechanical properties were also characterized.

## 2. Materials and Characterizations

### 2.1. Materials and Methods

The new type of 4D printing SMPs filaments based on the PLA were prepared by the Shenzhen Esun Industrial Co. Ltd. The essential details provided by the supplier are: the diameter (1.75 mm), density (1.23 g/cm^3^), flow index (5.8 g/10 min), and durability (4/10). The print failed several times due to the influence of the bed, print, and room temperature. The reason is that SMPs are temperature-sensitive materials and need a particular combination of process parameters. Printing SMPs under ordinary conditions is complicated, and the print mostly fails. Thus, it will affect the printing quality and may cause failures. At a higher bed temperature (55 °C), the initial layers begin to curl and fold, and the bed adhesion cannot resist a spontaneous deformation. At a shallow bed temperature (below 35 °C), the accumulation of the initial layers is minimal, and their bond strength with the top layers becomes weak, so the printing quality of the model was low. After a careful debugging and optimization, the model’s quality was higher for a bed temperature of 45 °C and a print temperature of 210 °C. A room temperature of 24 °C was maintained during the printing. The printer specifications with process parameters and print problems are provided in Appendix A. The printed filaments were used in fabricating the printed vascular scaffolds after the characterization of SMPs. The thermomechanical analysis of the SMPs was studied by dynamic mechanical analysis (DMA). The thermal properties and stability are observed by differential scanning calorimetry (DSC) and a thermogravimetric analysis (TGA). The tensile properties and shape relaxation tests at different temperatures were studied through Zwick tensile testing to develop the mathematical model and check the material behavior of the SMPs filaments in compressive unfolding and folding testing. All the specimens in the present study were printed with these parameters using a high-quality Flashforge Voxelab Aquila series 3D printer and its specifications are provided in Table 1.

### 2.2. Designing and Modeling of Vascular Scaffolds

**Figure 2:**
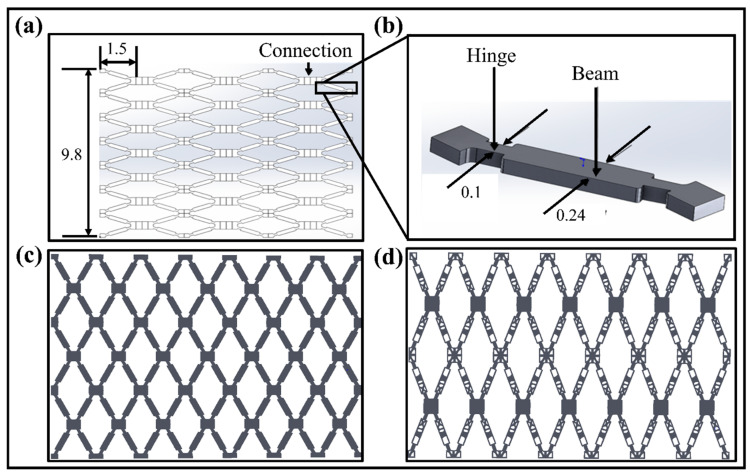
(**a**) An open symmetric vascular scaffold model and their dimensions (unfolded state). (**b**) A single unit structure of scaffold model with structural parameters. (**c**) Reconstructed original vascular scaffold structure in an unfolded state. (**d**) Optimized planer scaffold structure in unfolded state [All units are mm].

The vascular scaffold used in this paper is achieved by modifying and improving the vascular stent unit structure designed by Wu et al. (2008) [33]. It is re-modeled and re-calculated using SolidWorks to regenerate the original geometric model. The unfolded scaffold model with the highly symmetrical feature of the structure is shown in Figure 2a.

The structure of the scaffold unit cell with the parameters is illustrated in Figure 2b. The final original and optimized planer scaffold structures are given in Figure 2c,d. As per the medical standard of the People’s Republic of China for Implantable Medical Devices [34], the diameter of human blood vessels ranges from 3.26 mm to 4.16 mm, and the thickness of the blood vessel wall is 0.2 mm to 0.5 mm. The thickness of the stent allowed for an implantation at 0.025 mm to 0.177 mm. Here, to ensure the effectiveness of the scaffold design and the SMPs’ rebound characteristics, the reconstructed structure can effectively avoid an insufficient number of units in the tubular structure. The SIMP topological technique optimizes the structure with the high dimensional accuracy of the unfolded and folded structures. The reverse-engineering technique, performed in Geomagic Software, improves the dimensional accuracy of the optimized models. The optimization results with objectives and constraints are provided in Appendix B.

### 2.3. Characterization of Shape Memory Polymers (SMPs)

#### 2.3.1. Thermo-Mechanical Analysis of Shape Memory Polymers

Dynamic mechanical properties of a material can be obtained through DMA over a specific temperature range. The pattern under different dynamic loads and temperature responses can effectively analyze the rigidity and flexibility of a material. In the study of viscoelastic materials using DMA, the storage modulus, loss modulus, and loss angle (tan δ) were measured. The storage modulus indicates the elastic properties of the material as well as the energy absorption characteristics under the temperature effect. In contrast, the loss modulus refers to the energy dissipation characteristics of the material under the temperature effect (the viscosity characteristic) [35]. The loss angle represents the numerical ratio of the storage modulus to the loss modulus, also known as the damping factor [36]. The high storage modulus indicates that the material has a high elasticity and stiffness; a high loss modulus indicates that the material has a high viscosity and flexibility. The DMA test sample with dimensions 45 mm × 10 mm × 5 mm is prepared for analysis. The temperature ranges from 15 °C to 150 °C with a ramp rate of 2 °C/min using the tensile oscillatory mode, and a frequency of 1 Hz was applied. The type of loading was tensile–tensile.

#### 2.3.2. Thermal Analysis

The DSC analysis was performed on a DSC2500 Differential Scanning Calorimeter (US. TA) to assess the thermodynamic properties and crystallization. The heating and cooling rate was 10 °C/min with a temperature range of 30 °C to 200 °C. The thermal properties of the shape memory polymers (SMPs) used in this paper are studied by DSC to analyze the material stability, as well as the melting and glass transition temperature. The SMP filament with 10 mg was prepared and cut into small segments. In the DSC analysis, the second heating curve in the experiment is used to avoid the influence of factors such as the heat history during the material processing, and better and more accurate experimental results are obtained.

#### 2.3.3. Weight Loss and Decomposition Analysis

The weight loss and decomposition of the SMPs were analyzed through the TGA8000 Thermogravimetric Analyzer (Perkin Elmer, Waltham, MA, USA). The temperature range of 30 °C to 800 °C with a heating rate of 10 °C/min. The actual tendency of the material behavior is studied. The initial weight of 4 mg was taken and cleaned with nitrogen N2 for analysis.

#### 2.3.4. Morphology and Microstructural Analysis

The morphology and microstructural analysis of the SMPs matrix was observed through a field emission scanning electron microscope (Nova Nano SEM 450, FSEM, NETHERLANDS), and fracturing of the sample was done in liquid nitrogen. All the fractured surfaces were sprayed with a thin gold layer before testing.

#### 2.3.5. Tensile Properties and Stress Relaxation Tests

The tensile and relaxation tests were performed using a Zwick tensile instrument at different temperatures (25 °C, 35 °C, 45 °C, 55 °C, and 65 °C). The sample was prepared as per the ASTM-D638 standard. The load rate was set at 2 mm/min in the experiment. Stress relaxation testing is the gradual reduction of stress on the polymer materials under constant strain conditions. The test sample was prepared according to the GB/T 11546.1 standard to meet the manufacturer’s requirements for the stress relaxation test. The machine was heated initially for five minutes for the uniformity to ensure a high test accuracy. The speed was 2 mm/min, and the strain was maintained at 100% for 1800 s. The relaxation data for 500 s and 1800 s were recorded, respectively. The tensile test for the elastic modulus was conducted using SHIMADZU tensile machine at 25 °C. The strain versus stress curves were determined by finding the F−Δl curve, and then the elastic modulus was calculated.

#### 2.3.6. Shape Memory Properties

The shape memory behavior of SMPs was characterized through the 4D-printed circular tube in a hot water bath. The tube with a temporary shape was put in hot water at a temperature higher than the glass transition temperature of SMPs. A camera was used to record the whole shape recovery process. The influence of the recovery temperature and tube thickness on the shape memory behavior was also determined. This unique feature makes SMPs a potential candidate for utilization in vascular scaffolds.

### 2.4. Performance Analysis of Vascular Scaffolds

The structural design of vascular scaffolds plays a vital role in treating a vascular implantation. The distribution of trenches shows that the application of topology optimization technology to the design of biodegradable scaffolds is still relatively small. The mechanical properties of the vascular scaffold are evaluated under radial compressive loading until the failure occurs. Due to the precision limitations of the traditional FDM (fused deposition modeling), an advanced 3D printer is used to fabricate the vascular scaffold structure by SMPs. The SL-AM service is provided by Jiangsu Yuyuan Zhifeng Product Design Co., Ltd. The LCD (Liquid Crystal Display) manufacturing technique, a kind of high precision AM method, is performed on Photon Mono X Pro (Shenzhen ANYCUBIC Technology Co., Ltd., Shenzhen, China). The shape memory behavior is also determined through the self-folding property to check the functionality of the 4D printed vascular scaffold at the recovery temperature, which can be adopted in the human body.

## 3. Results and Discussion

The heat shape memory polymers (heat-SMPs) were deeply investigated based on the appropriate transition temperature with superb thermal, mechanical, and shape memory properties. Various characterizations showed that the present SMPs material could be perfectly adaptable in fabricating the vascular scaffold for a vascular implantation. The radial shape memory properties were assessed through a tube structure at different recovery temperatures and tube wall thicknesses. Finally, a novel porous vascular scaffold model was proposed and fabricated using the present heat-SMPs. Their flexibility and mechanical properties were analyzed through compressive, three-point bending, and shape memory experiments in water, which showed the superiority of the proposed scaffold.

### 3.1. Heat-Shape Memory Polymers

#### 3.1.1. Thermo-Mechanical Analysis of Shape Memory Polymers

**Figure 3:**
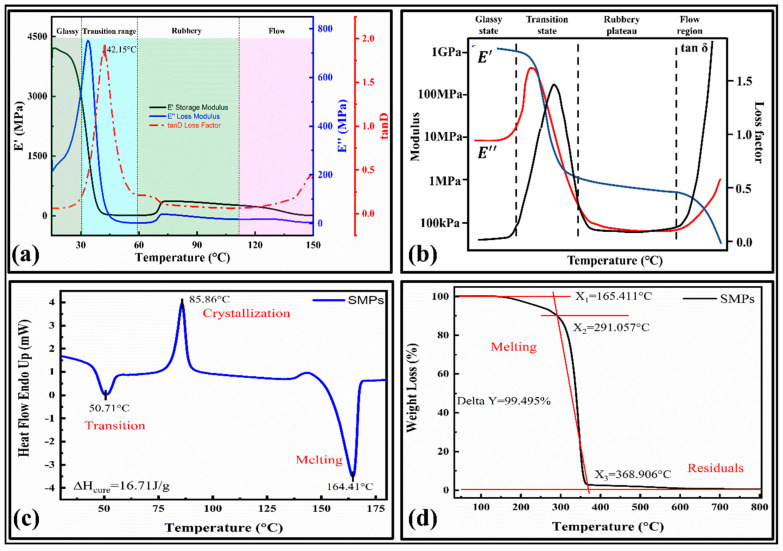
Characterizations of shape memory polymers; (**a**) DMA curves for temperature versus storage modulus, loss modulus, and loss factor; (**b**) a typical DMA curve and related terminologies; [37] (**c**) DSC curve; (**d**) TGA curve.

It provides temperature in phase changes and verifies their shape memory behavior [33]. The storage and loss modulus versus the temperature curves are provided in Figure 3a. The main results were compared with the typical DMA curve, which showed a high relevancy. The curve consists of four main phases [37]: (1) a glassy state, (2) glass transition state, (3) rubbery plateau, and (4) flow state, as shown in Figure 3b.

The glass transition temperature for the present SMPs is 42.15 °C. It can be observed that the initial value of the storage modulus (E’) at 15 °C is 4000 MPa, which is about 20 times that of the loss modulus (E’’), which indicates that the initial state of the material is the glass state in range of 15 °C–32 °C, and the stiffness is significant (tan δ = 0.07375). As the temperature increases, the material’s storage and loss modulus decline around 40 °C, indicating that the material changes from a glassy state to a different state. Finally, the material will flow as a liquid. The storage modulus quickly dropped within the range (32 °C to 56 °C) and the material showed a lower modulus above the glass transition temperature, demonstrating shape memory behavior. The loss modulus also decreases rapidly within the region (32 to 50) °C and has lower values at the higher temperature. In the viscoelastic material test, the stress has a hysteresis effect on the temperature, so the change in the strain lags behind the stress, and the actual map also has a phase difference.

#### 3.1.2. Thermal Analysis

The phase change in the material can regulate the shape memory behavior. Hence, the thermal and crystallization/melt behavior is determined by the DSC results, as illustrated in Figure 3c. It can be observed that the first heat absorption range of the material is from 40 °C to 60 °C, and at 50.71 °C, the heat flow curve of the material reaches the first peak, which indicates that the material undergoes a glass transition (T_g_), which is close to the DMA results. The peaks in the curves represent the crystallization and melting of the SMPs. We can see that the crystallization and melting temperatures are 85.86 °C and 164.41°C, respectively. After calculating the area of the crystallization process, the curing heat released by the crystallization Δ*H_cure* = 16.71 J/g. This phenomenon also shows that the SMPs used in this paper have semi-crystalline characteristics. The crystallinity was determined by,

(1) Relative crystallinity=[ΔHmΔH0m]×100

Here, $\Delta {H^{0}}_{m}$ is the crystalline melting enthalpy obtained from the crystalline endothermic melting curve (found to be 16.71 J g^−1^ in the present work) and $\Delta H_{m}$ is the melting enthalpy estimated from the crystalline melting peaks of the SMPs. The crystallinity determined for PLA was 38.5%. The remaining part is amorphous. It has an inverse relationship with the permeability of the polymer composites. Their effect on the shape fixity ratio will be adequately studied in the future by experiments. The nozzle temperature for the 3D printer is selected based on the melting temperature. Therefore, the nozzle temperature for the SMPs is around 200 °C.

#### 3.1.3. Weight Loss and Decomposition Analysis

The thermogravimetric (TGA) curves of the 4D printed SMPs are shown in Figure 3d. The SMPs are initially decomposed (melted) at 165.411 °C (X_1_), and the temperature at the highest thermal decomposition rate was 291.057 °C (X_2_). The SMPs showed a good thermal stability at a peak temperature of 165.411 °C. With a further increase in the temperature above this point, the first decomposition (pyrolysis action) occurs. Finally, at the end of the thermal decomposition, the temperature was 368.906 °C (X_3_). The SMPs are completely decomposed at around 370 °C, with the residual weight percent being around 0.505% (Delta Y = 99.495%). The temperature required to degrade the SMPs to 50% weight loss is 340.310 °C. Based on the thermal analysis, the print temperature for the SMPs was 180 °C–220 °C.

#### 3.1.4. Mechanical Properties of SMPs

**Figure 4:**
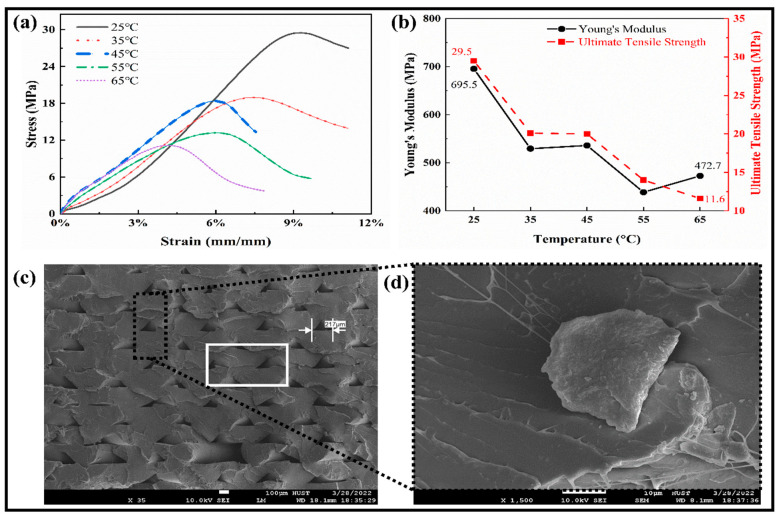
Tensile testing measurements. (**a**) The tensile stress–strain relationship at different elevated temperatures. (**b**) The Young’s modulus and Ultimate tensile strength of SMPs at different temperatures. (**c**) SEM image of the deposited layers in the fractured region at room temperature. (**d**) The magnified image of the highlighted part.

The mechanical properties were studied through tensile testing for the SMPs specimens, as shown in Figure 4a. All the curves followed a similar trend for the shape memory polymers at different temperatures. The relationships between Young’s modulus and the ultimate tensile strength against the temperature are shown in Figure 4b. With the increase in temperature, the ultimate tensile strength of the SMPs is reduced from 29.5 MPa to 11.6 MPa. At a low temperature (25 °C), the modulus and strength limits are higher and have lower values at a high temperature. In the temperature range (35–45) °C, it becomes stable, which means that the transition temperature lies in this region, which is close to the human body temperature and is adaptable to the human body in vivo. So, it can be used to fabricate a medical implantation such as blood vessel scaffolds, etc. By taking 45 °C (close to T_g_) as the boundary, the “high-temperature zone” and “low-temperature zone” are separated. As the temperature rises, the elastic modulus of the polymer decreases. We can select the appropriate strain value to estimate Young’s modulus in the elastic range. In the stress–strain curve, the stress peak value of the high-temperature test curve decreases with the increase in temperature and vice versa. The above results show that the peak values are relatively similar for the temperature curves (35 °C and 45 °C).

Furthermore, by combining with the theory of SMPs, for the material that underwent the glass state, viscoelastic state, and high elastic state, it can also be concluded that the glass transition temperature of the material should be around 45 °C. The SEM images of the fracture sample during the tensile test are shown in Figure 4c,d. The small layers gaps show the weak deposition of the layers, which significantly affects the performance measures. The gap in a rectangular box shows the dislocation of the layers and the flexible nature to resist for a more extended period.

#### 3.1.5. Relaxation Tests

**Figure 5:**
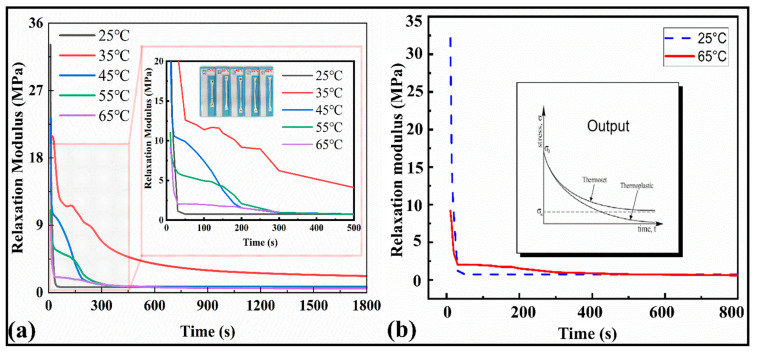
(**a**) Stress relaxation curves at different temperatures versus the time for the range of 1800 s and 500 s. (**b**) Stress relaxation curve at 25 °C and 65 °C and stress relaxation curve for ideal thermal polymers and their classification.

The relaxation modulus results for the SMP are depicted in Figure 5a,b. It can be observed from Figure 5a that as the temperature increases, the peak values of the modulus decrease from 35 MPa to 9 MPa, and finally they tend to be at a stable value of 0.54 MPa over time. The magnified plot shows the variation among the relaxation moduli at different temperatures. The modulus curves turn to a stable value σ∞ after 800 s until the end of the cycle (1800 s), exhibiting themselves as thermosets. Similarly, the relaxation time at 25 °C is sharp (less than 100 s), while at 65 °C, the relaxation modulus tends to equilibrium at about 1500 s (Figure 5b). This shows that with the increase in temperature, the viscosity flow characteristics of the material appear, and a hysteresis effect on the modulus occurs. As a result, the internal mechanical equilibrium of the material can be maintained for a longer time. The maximum modulus at room temperature was around 34 MPa.

In the subsequent description of the mathematical model, the above data can be processed for modeling and analysis to extract the parameters of a double logarithmic curve suitable for the Maxwell multivariate constitutive model. The relaxation curves indicated (Figure 5b) that the present polymers belong to the thermoset family. The stress and strain relationship results are manifested with the thermomechanical properties of the SMPs. The average elastic modulus of the SMPs material (five specimens were tested) was 861.309 MPa.

#### 3.1.6. Unfold Shape Memory Properties

Generally speaking, the shape recovery behavior can be characterized by the shape recovery ratio (R_r_) in a radial direction and unfolding state. These two parameters refer to the capabilities to maintain the temporary shape and recover the original shape. The process is as follows: a circular tube was first deformed in a radial direction to an intermediate shape upon loading at temperatures (65 °C, 75 °C, and 85 °C) higher than the glass transition temperature (T_g_) of the SMPs in hot water. The printed tube with a temporary shape was put in hot water at a temperature higher than the glass transition temperature of the SMPs.

The deformation temperature (T_d_) was taken at 90 °C for each sample, and the samples were placed for around three minutes. The temporary shape was fixed, cooled to room temperature, and released the external load. The influence of the recovery temperature and tube thickness on the shape memory behavior was observed.

The recovery ratio was computed by the following Equation (2).

(2) $$R_{r}\left(\%\right)=\left(\frac{h_{t}-h_{i}}{h_{0}-h_{i}}\right)\times 100\%$$

**Figure 6:**
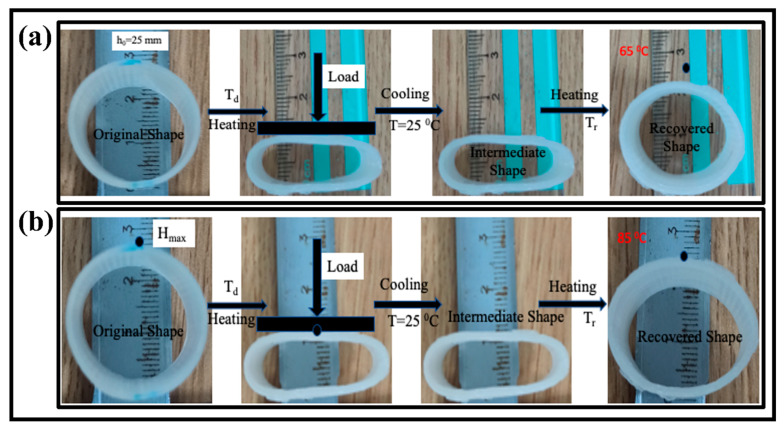
The shape memory cycle of the 4D printed tube structure; (**a**) shape recovery in response of 65 °C hot water with tube thickness of 2 mm; (**b**) shape recovery in response of 85 °C hot water with tube thickness of 2 mm.

**Figure 7:**
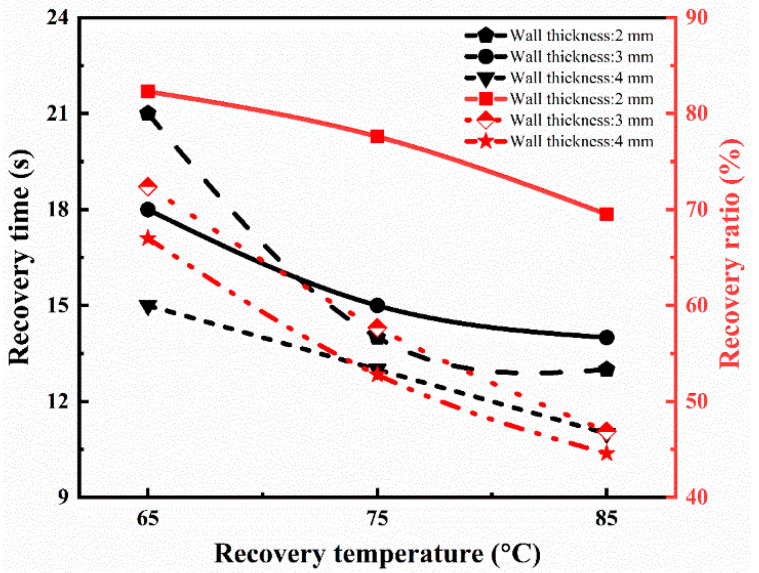
Recovery ratio and recovery time at different recovery temperatures for 4D printed tube during the shape memory process.

In the above equation, *h*_0_, *h_i_*, and *h_t_* are the cross-sectional height of the tube at the initial, temporary, and recovered time (t), respectively. The whole recovery cycle of the printed tube with a tube thickness of 2 mm at the recovery temperature of 65 °C is shown in Figure 6a. The tube sample was radially recovered to its initial shape in 21 s. The recovery ratio turned out to be 82.3%. Figure 6b shows the recovery process with the time variation at a recovery temperature of 85 °C with a similar thickness of 2 mm. The SMPs tube regained its initial shape in 13 s, and its recovery ratio is 69.5%, which is higher than the previous report [38]. Similarly, the effect of the recovery temperature (65 °C, 75 °C, and 85 °C) and thickness (2 mm, 3 mm, and 4 mm) on the shape memory behavior was investigated. The detailed analysis of the recovery ratio and recovery time is illustrated in Figure 7. It is concluded that the recovery rate increases with the increase in the recovery temperature. On the other hand, the recovery ratio decreases with the rise in the recovery temperature. At a lower recovery temperature (65 °C) with a thickness of 2 mm, the recovery ratio was 82.3%, while at a higher temperature (85 °C), the recovery ratio was 69.5% for the same thickness. It followed the trend of previous results and possible reasons [39].

During the shape fixity, the axial deformation is maintained by the molecular chain of the polymer and its contraction upon the applied force [39,40]. The chain can change itself according to the external stimuli above T_g_ for the stated thickness.

As the wall thickness increases (3 mm), the recovery ratio is 72.4% at a low recovery temperature, which is lower than the wall thickness of 2 mm for the same recovery temperature. If we further increase the tube’s wall thickness (4 mm), the recovery ratio (67.0%) is even less at a lower temperature. It means that the recovery ratio decreases with the increase in the wall thickness of the tube. It means that the recovery ratio is maximum at a higher recovery temperature for the wall thickness of 2 mm compared to others. Furthermore, the recovery ratio decreases as the thickness increases, but the rate increases to regain its shape quickly, and no further change is detected. The results are similar to the report [39,41], which showed the authenticity of our conclusions.

### 3.2. Performance Analysis of Proposed Vascular Scaffolds

#### 3.2.1. Radial Compressive Performance

**Figure 8:**
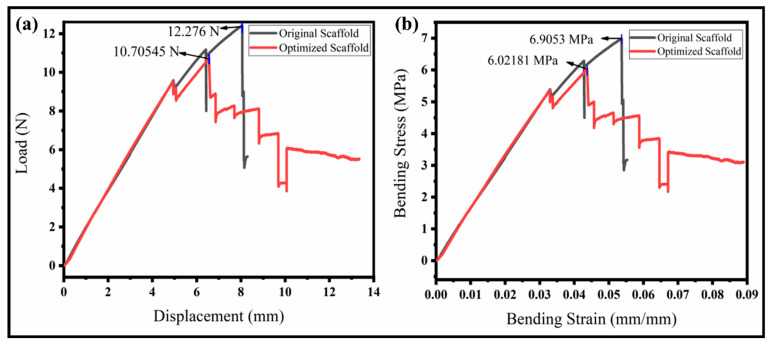
Radial compressive and three-point bend testing of the original and our optimized scaffolds. (**a**) Compressive stress–strain curve; (**b**) flexural stress–strain curve.

The scaffold can be subjected to compressive loading during the implantation, so its compressive strength should be evaluated. The sample was printed according to the ASTM D2412 standard. The compressive stress–strain curve is shown in Figure 8a. The mechanical properties of the vascular scaffold are evaluated under radial compressive loading. The compressive test of the vascular scaffold was carried out in three stages: (1) the non-linear initial compression stage, which is mainly related to when the plate has not yet come in contact with the scaffold; (2) the elastic deformation stage, in which the upper plate and the scaffold are in full contact; and (3) the plastic deformation stage; as the deformation continues to increase, the scaffold undergoes a plastic deformation until the deformation reaches a specific limit, the individual connecting ribs are in contact with each other, and, further, no deformation occurs in the scaffold. As a result, the resistance increases non-linearly. The slope has increased to a certain extent because the lower surface of the structures and the contact surface of the lower pressure plate are no longer in line contact, so the slope increases and meets the principle of elastic deformation.

The peak loads for the original and proposed scaffolds were achieved at a displacement of 25.72 mm and 26.49 mm, respectively. Further, the increase results in the plastic deformation and the initial shape is not regained. The main reason is that particular supporting ribs are broken initially, and more connecting ribs further prevent the complete rupture of the structure. An optimized structure looks more stable than the original one due to holes (pores) in the ribs that will divide the pressure uniformly throughout. The pressure is transformed into a whole structure uniformly. It demonstrates the actual strength of the vascular scaffold. The compressive strength of our optimized scaffold was about 0.092 MPa (higher than the original), which is enough for the application of a vascular scaffold implanting into the body as per the medical standard [34], as shown in Figure 8a. The maximum load of the optimized model is close to 50 N, which is 3.5 N, higher than the original vascular structure, which is higher than the results achieved in a previous study [42]. The curve followed the elasticity–plasticity principles. There is a non-linear relationship between the reaction force and displacement, and the failure of the sample occurs in the plastic region by further applying the load. The optimized model can withstand about an equal displacement while requiring a higher ultimate reaction force than the original model. The optimized structure’s yield point and proportional limit are also more excellent than the original structure. Our proposed model’s compressive strength and modulus are higher than the original model. Similarly, the model would generate more minor cracks and keep the model integrated, while in the original model, the separated parts would fall.

#### 3.2.2. Three-Point Bending Properties of the Vascular Scaffold

The flexibility of the vascular scaffolds was evaluated through a three-point bend test of the scaffolds at room temperature. The sample properties and attributes, such as the thickness, width, and pivot span, are 4 mm, 10 mm, and 60 mm, respectively. The bending results to evaluate the resistive load and flexural strength or bending stress is illustrated in Figure 8b. The resistive load to deformation varies with time. Both scaffolds show a high resistance to bend at the early stages of loading. The deformation increases gradually with the sufficient applied load for yielding until it loses the point support and no more deformation occurs. The maximum load for the original scaffold is high (12.28 N) compared to the optimized scaffold (10.71 N), which is higher than the literature [43]. However, the interesting fact is that the original scaffold suddenly failed upon reaching the maximum load resistance. The optimized scaffold shows a high stability even after the peak resistance. The optimized structure is bettered by having a minimum weight or compliance. The moment of resistance ability of the optimized scaffold is better than the original due to a certain toughness and durability around the body temperature. Therefore, the specimens did not break but just lost their stability. The flexural strength, also known as the bending stress, is observed, as illustrated in Figure 8b. The flexural strength of the original scaffold is higher than the optimized scaffold, but it failed suddenly to reach the peak value. This means the stress distribution in the original structure is not uniform, and peak stress occurs at the center, which resists internally but fails and is broken. We can observe from the interesting curve for the optimized scaffold that the flexural stress is less but very close to the original one. The stress distribution is also uniform, resulting in a higher internal resistance to bending. The stress difference for both scaffolds is between 10–15%, showing a good compromise for the analysis of the minimum weight or compliance ratio. It has been concluded that the proposed scaffold performs well in a complex environment.

#### 3.2.3. Functionality of the Vascular Scaffold

**Figure 9:**
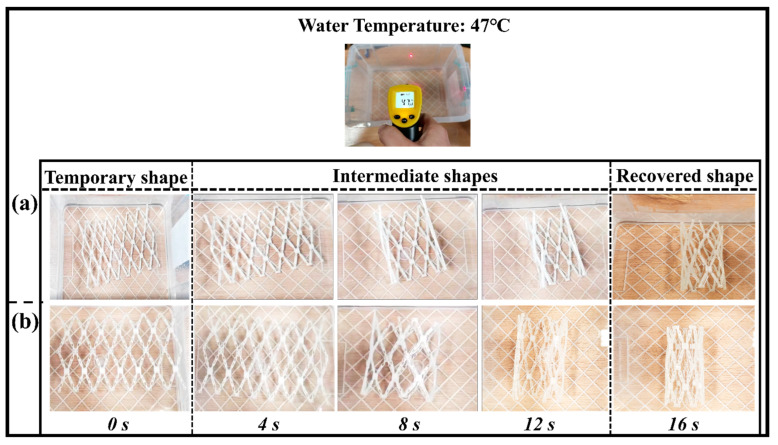
Shape memory behavior of blood vessel scaffold at a recovery temperature of 47 °C. (**a**) Shape recovery of the original scaffold structure; (**b**) shape recovery of an optimized scaffold structure.

The vascular scaffold has an outstanding radial recoverability and excellent self-folding property for the shape memory behavior. This test is basically to check the functionality of the shape memory polymers in the application of a vascular implantation. The initial shape of the vascular scaffold is recovered circumferentially, which was subjected to a load (can be manual) to an unfolded position as a plate. The shape memory behavior of the original and optimized structure is demonstrated in Figure 9a,b.

The transition temperature of the shape memory polymer is nearly close to the human body temperature. Therefore, it is feasible to choose a recovery temperature greater (10 °C) than the transition temperature of the SMPs. The recovery temperature for the present experiment is 47 °C, which is less than the previous report [44]. The unfolded sample regains the near shape of the vascular tube structure at this low recovery temperature at a time of 16 s. The shape recovery ratio (R_r_) of the vascular scaffold which was calculated was around 98% (close to 100%). The optimized structure has higher overturning and stronger structure compactness. The SMPs can also be used as a functional material in other applications (mechanical grippers, stents fabrication, actuators, etc.) due to the recovery at different temperatures. These results are closed with the previous studies reported by other researchers [19].

## 4. Performance Comparison with Previous Models

This study focuses on designing a new class of scaffolds with good mechanical properties suitable for treating vascular restenosis in vascular tissue engineering. For this reason, a new kind of vascular scaffold was designed, optimized, and mechanically tested in our laboratory. Based on the unique properties of thermal SMPs, we have successfully developed the original and optimized porous vascular scaffolds without extra support. The characterizations showed excellent mechanical properties and the stability of the developed vascular scaffolds, which are compared and verified with the standard arterial clinical requirements and previously developed vascular scaffolds. The proposed scaffold is compared with some recent studies, as provided in Table 2.

The transition temperature of our proposed scaffold is better and is close to the body temperature. In other studies, it is either lower or significantly higher than the normal temperature, which may damage the soft tissues. The mechanical properties and shape recovery are superior to that provided in [45]. The glass transition temperature (42.15 °C) is lower than the previous work (65 °C), which is closer to the human body temperature and may be considered safe for human tissues. The mechanical properties of our scaffold in terms of the tensile, compression, and bending test are better than the previous studies. In most studies, the researchers focused on either the tensile or compressive properties. We have also compared these with the medical standards [34] for human great saphenous veins and observed that the results are in the eligible range for clinical use, which is already discussed in the previous section. Therefore, it is concluded and verified that the thermal shape memory polymers (SMPs) and vascular scaffolds developed in the present study can meet the clinical strength requirements for artificial vascular scaffolds.

## 5. Conclusions and Outlook

In this research, the SMPs were the primary focus for the vascular scaffold based on the required mechanical and thermal properties per the medical standards. Various characterizations showed that the present SMPs could be perfectly adaptable in the scaffold implantation in vascular soft tissue. The temperature range (35–45) °C becomes stable as the transition temperature (43°C) lies in this region, which is close to the human body temperature and is adaptable to the human body in vivo. This is also verified by the DSC results and describes the phase transition (transition, melting, and crystallization) of the SMPs. Therefore, it can be used to fabricate a medical implantation, such as blood vessel scaffolds in the present case. The recovery behavior was studied through the radial compression unfolding and folding conditions. It was concluded that the recovery rate increases with the increase in the recovery temperature. On the other hand, the recovery ratio decreases with the rise in the recovery temperature. At a lower recovery temperature with a thickness of 2 mm, the recovery ratio is 82.3%, while at a higher temperature, the recovery ratio is 69.5% for the same thickness. As the wall thickness increased (3 mm), the recovery ratio is 72.4% at a low recovery temperature, which is lower than the wall thickness of 2 mm for the same recovery temperature. If we further increase the tube’s wall thickness (4 mm), the recovery ratio (67.0%) is even less at low temperatures. It is concluded that the recovery ratio decreases with the increase in the wall thickness of the tube. Based on the classification and properties of the shape memory polymer and their stress relaxation performance, it can be regarded as a “thermoset” polymer. The designed scaffold was optimized through topology optimization, resulting in a porous scaffold. The compressive and bending results showed that the optimized structure is more stable than the original due to the pores in the ribs, which can uniformly divide and transfer the pressure in a unit and final structure. The tensile strength and modulus of our proposed model are higher than the original model as well as the recent literature. Similarly, the model would generate more minor cracks and keep the model stable. The structure regains its initial shape of the vascular tube structure in a recovery time of 16 s. The shape recovery ratio of the vascular scaffold was around 98%. The deformation increases gradually with the sufficient applied load for yielding until it loses the point support and no more deformation occurs. The optimized scaffold shows a high stability even after the peak resistance. The moment of resistance ability of the optimized scaffold is better than the original due to a certain toughness and durability around the body temperature. It has been concluded that the proposed scaffold is more flexible and performs well in a complex environment.

In the future, our proposed scaffold will be 4D printed at the nano/micro scale using the stated SMPs and will be biologically assessed through an implantation with the proper protocol. Various biological tests will be conducted before the implant and we will check the blood compatibility.

## Figures and Tables

**Table 1:** Main specification of Flash forge Voxelab Aquila.

Specifications	Value
Nozzle diameter	0.4 mm
Precision	±0.2 mm
Bed temperature	≤120 °C
Nozzle temperature	≤250 °C
Maximum print size	220 mm × 220 mm × 250 mm
Print speed	30–60 mm/s (Max.180 mm/s)
Slicing software	Cura, Simplify, Voxelmaker

**Table 2:** Comparison of vascular scaffolds properties.

T_g_ *, °C	TS *, MPa	YM *, MPa	T_crys_ *, °C	T_m_ *, °C	RM * (25 °C), MPa	R_r_ *, %	BS *, MPa	Material
42.1	29.5	695.4	85.86	164.4	34	98 (47 °C)	6.02	PLA-SMPs (This study)
66.4	21.3	267	122.9	167.8	41	90 (65 °C)	4.36	Shape memory PLA [45]
33	19.3 ± 0.9	743 ± 24	-	-	-	82 (37/40 °C)	-	Shape memory polyurethane nanocomposites (PCLAU/Fe_3_O_4_) [46]
51.56	6.93	102.23	56.7	60.33	-	89.8 (70 °C)	-	Shape Memory PEG-PCL [47]
-	7.8	20	-	-	-	-	-	Shape memory PLA [48]
-	25	600	-	-	-	-	-	heparin-grafted poly(ester-urethane)urea/gelatin (PU75-E/N-Hep) [49]

* Notations: T_g_ = transition temperature, TS = tensile strength, YM = Young’s modulus, T_crys_ = crystallization temperature, T_m_ = melting temperature, RM = relaxation modulus, R_r_ = recovery ratio, BS = bending strength.

**Figure A1:**
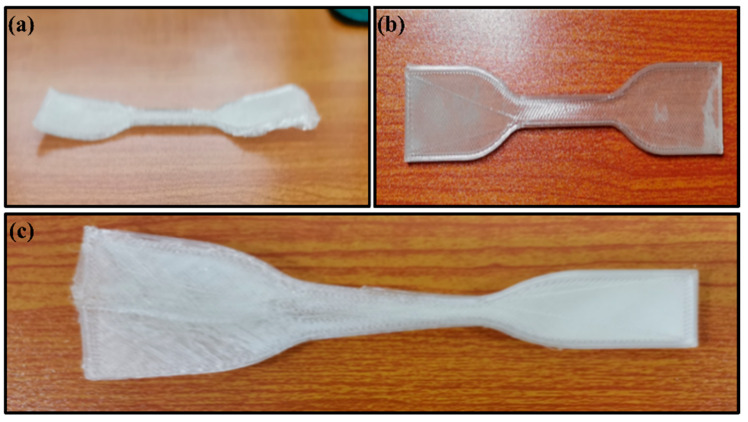
3D printing at different temperatures (**a**) bed temperature at 55 °C (**b**) bed temperature at 45 °C (**c**) bed temperature at 35 °C.

**Figure A2:**
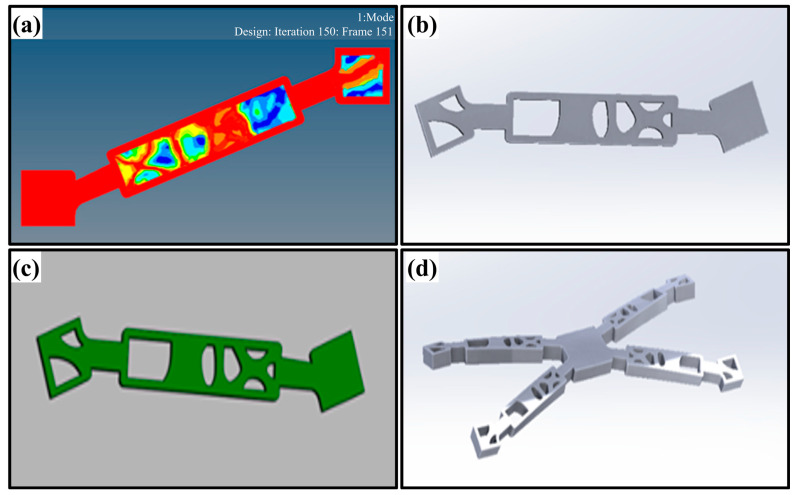
Optimize unit structure and reverse engineering; (**a**) the optimized structure in Hyper mesh, while the red area is the retained area, and the rest is the material area that can be removed for optimization; (**b**) the reversed engineered structure generated by GEOMAGIC; (**c**) is the comparison of the optimized model (stl format) and the reversed engineered model (stp format), the greener region means more better structure. (**d**) a unit model of the proposed scaffold.

## Data Availability

Not applicable.

## Appendix A. 3D Printing of the Vascular Scaffold

An advanced Flashforge Voxelab’s Aquila 3D printer was used to print the models. When the temperature is set to 55 °C, the initial layer of printing begins to curl and wrinkle, and the adhesion of solid glue is unable to resist the spontaneous deformation (Figure A1a). The molding quality is high when the bed temperature is set to 45 °C (Figure A1b). When the bed temperature was reduced to 35 °C, the initial layer temperature accumulation was low. With the printing progress, the top layer temperature continued to increase, and the model shifted from the top (Figure A1c).

After additional tests for different nozzle temperatures, platform temperatures, and auxiliary boundaries, the parameters and their chosen levels are provided in Table A1.

**Table A1:** Process parameters and their values for process optimization.

Infill Pattern	Room Temperature (°C)	Nozzle Temperature (°C)	Bed Temperature (°C)	Secondary Boundaries	Remarks
100%	24	210	55	No	The initial layer is curls
100%	24	210	55	Yes	
100%	24	210	45	No	Higher print quality
100%	24	210	45	Yes	
100%	24	210	35	No	Top-level offset
100%	24	210	35	Yes	

## Appendix B. Structural Topology Optimization of Vascular Scaffold Based on SIMP Method

The SIMP (solid isotropic materials with penalization) topological technique optimized the original scaffold structure with a high dimensional accuracy of unfolded and folded structures. The dimensional accuracy of optimized models is improved by the reverse engineering technique performed in GEOMAGIC Software. The parameters of the Prony series determined in by the viscoelastic modeling in the previous chapter are imported into HyperWorks, and then the load is applied. To accurately ensure the optimization, the model is divided into the design domain and the non-design domain. The red area is considered the design domain (optimization area) and the grey area is a non-design domain. A regular tetrahedral meshing is applied for structural division, and the number of units and nodes is also determined.

According to the topology optimization, the objective is to minimize compliance, and the mathematical model can be expressed as Equation (A1).

Objective:

(A1) $$\min C=\left\{\begin{array}{c} \begin{array}{c} =U^{T}KU\\ =\sum_{e=1}^{N}u_{e}^{T}k_{e}u_{e} \end{array}\\ \sum_{e=1}^{N}{\left(x_{e}\right)}^{p}u_{e}^{T}k_{0}u_{e} \end{array}\right\}$$

Subject to:

(A2) $$\begin{array}{c} \begin{array}{c} \frac{V\left(x\right)}{V}=f\\ KU=F \end{array}\\ K_{e}={\left(x_{e}\right)}^{p}k_{0}\\ M_{1}=\frac{EI_{s}}{R}\\ 0\leq x_{min}\leq x\leq 1 \end{array}\}$$

Here, K represents the stiffness matrix, U represents the displacement matrix, $U^{T}$ represents the transpose matrix of the displacement matrix; $u_{e}^{T}$, $u_{e}$, and $k_{e}$ represents the transpose matrix of the unit cell stiffness matrix, the cell stiffness matrix, and cell displacement matrix, respectively; f Represents the volume fraction; R represents the inner diameter of the vascular scaffold, $x_{min}$ represents the minimum design variable (non-zero constant), avoiding the matrix singularity of the computational process.

The optimized HyperWorks model in STL format is imported into GEOMAGIC software for reverse engineering for further analysis. Volume evaluation in Geomagic Color Bar under the OPTISTRUCT module can demonstrate the feasibility and accuracy of reverse engineering.

Figure A2a shows the unit structure optimized by the HyperWorks OPTISTRUCT module and Figure A2b represents the optimized model for reverse engineering in GEOMAGIC software. The green color of the unit structure indicates that the reverse engineering model matches the STL file to a high degree, and the reverse modeling accuracy of the corresponding model is also high, according to the scale shown in Figure A2c. The final optimized scaffold structure is obtained by assembling the unit structures, such as the partially folded structure shown in Figure A2d. The final model achieved in folding and unfolding state optimized by the stated SIMP topology optimization are already provided in Figure 1 and Figure 2 (in the main text), respectively.
